# Application of Air-Coupled Ground Penetrating Radar Based on F-K Filtering and BP Migration in High-Speed Railway Tunnel Detection

**DOI:** 10.3390/s23094343

**Published:** 2023-04-27

**Authors:** Yang Lei, Bo Jiang, Guofeng Su, Yong Zou, Falin Qi, Baoqing Li, Feiyu Jia, Tian Tian, Qiming Qu

**Affiliations:** 1 Infrastructure Inspection Research Institute, China Academy of Railway Sciences, Beijing 100081, China; pjiang@rails.cn (B.J.); qifl@rails.cn (F.Q.); tiantian@rails.cn (T.T.);; 2 Southwest Jiaotong University Research Institute (Chengdu) Co., Ltd., Chengdu 610036, Chinabqli@my.swjtu.edu.cn (B.L.); 3 Beijing ZhuoRuiXinDa Technology Co., Ltd., Beijing 101599, China

**Keywords:** high-speed railway tunnel, air-coupled GPR, F-K filtering, BP migration, defect census

## Abstract

As the number and length of high-speed railway tunnels increase in China, implicit defects such as insufficient lining thicknesses, voids, and poor compaction have become increasingly common, posing a serious threat to train operation safety. It is, therefore, imperative to conduct a comprehensive census of the defects within the tunnel linings. In response to this problem, this study proposes a high-speed railway tunnel detection method based on vehicle-mounted air-coupled GPR. Building on a forward simulation of air-coupled GPR, the study proposes the F-K filtering and BP migration algorithms based on the practical considerations of random noise and imaging interference from the inherent equipment. Through multi-dimensional quantitative comparisons, these algorithms are shown to improve the spectrum entropy values and instantaneous amplitude ratios by 4.6% and 11.6%; and 120% and 180%, respectively, over the mean and bandpass filtering algorithms, demonstrating their ability to suppress clutter and enhance the internal signal prominence of the lining. The experimental results are consistent with the forward simulation trends, and the verification using the ground-coupled GPR detection confirms that air-coupled GPR can meet the requirements of high-speed railway tunnel lining inspections. A comprehensive GPR detection model is proposed to lay the foundation for a subsequent defect census of high-speed railway tunnels.

## 1. Introduction

In China, over 16,798 tunnels have been constructed, with 3631 of them dedicated to high-speed rail transportation [1]. These tunnels play a vital role in ensuring smooth and safe operations for high-speed railways. However, the presence of lining cracking and internal lining defects caused by various factors can compromise the structural integrity of the tunnels and disrupt the train operations. Therefore, it is imperative to conduct a comprehensive, rapid, and accurate health census of the tunnel linings to ensure the safety and normal functioning of the high-speed railways.

Various techniques, including impact-echo, the microtremor method, infrared thermography, ground penetrating radar (GPR), and ultrasonic tomography, have been commonly used for detecting internal defects in tunnel linings [2,3,4,5]. Studies have shown that these methods can effectively detect defects in tunnel linings and achieve satisfactory detection results [6,7,8]. The combination of multiple techniques [9,10] can especially enhance the identification and localization of the defects. Among these methods, GPR, particularly vehicle-mounted GPR [11], is more efficient and widely used in high-speed railway tunnel inspections [12]. However, the application of ground-coupled GPR is limited by the antenna-to-lining distance (within 20 cm) [13] and the need to avoid contact with the railway overhead line and complex control system, making it difficult to achieve a rapid and efficient inspection of the tunnel linings during the maintenance periods. Therefore, vehicle-mounted air-coupled GPR, which offers advantages such as rapid, long-distance, and continuous inspection, has gradually been applied in tunnel inspections. However, current applications are mainly focused on road tunnels [14,15,16] and pavement inspections [17] using high-frequency antennas that cannot meet the requirements for the lining detection depth. The railway vehicle-mounted GPR detection system developed by the research team led by Professor Zan Yuewen at Southwest Jiaotong University achieved some success in detecting lining defects over long distances [18,19]. At present, the radar data filtering of the vehicle-mounted GPR system mainly adopts the bandpass filtering method, and the filtering of interference, such as random signals, needs to be further improved. In addition, due to the masking interference of the metal catenary and lining surface ancillary facilities of the high-speed railway tunnel on the deep signal of the lining, the judgment of the internal defects of the lining is affected. Therefore, we need to further study the interpretation and detection accuracy of the GPR images.

In this paper, based on the vehicle-mounted air-coupled GPR system, it first forward simulates the working conditions of the different detection distances and proposes an F-K filtering algorithm based on the butterfly filter and a BP migration algorithm in combination with the interference situation existing in the high-speed rail tunnel. Using a multi-dimensional quantitative comparison, the algorithm can suppress clutter and improve the signal prominence inside the lining. Then, the applicability of vehicle-mounted air-coupled GPR to detect high-speed rail tunnels and the effectiveness of the algorithm are verified through actual detection. Finally, compared to the ground-coupled GPR detection data, a comprehensive GPR detection method that meets the applicability and accuracy of the high-speed rail tunnel health census is proposed.

## 2. Forward Modeling and Algorithm Improvement of Air-Coupled GRP Tunnel Detection

### 2.1. Forward Modeling of the Different Detection Distances

The article describes the application of a software called GprMax, which was specifically designed for simulating GPR forward modeling. The software is based on the FDTD principle for simulating electromagnetic wave propagation, and it allows for different types of antennas to be used depending on the specific detection needs. Due to the complex situation of the lining of high-speed railway tunnels, GPR forward modeling simulations typically start with simple models. Therefore, this article designed a horn-shaped radar antenna model based on the actual situation of a high-speed railway tunnel and simulated a simulation model for the lining detection at different detection distances and cavity sizes. The design parameters are listed in Table 1 following the querying of the medium-related electromagnetic parameters [20,21].

**Figure 1:**
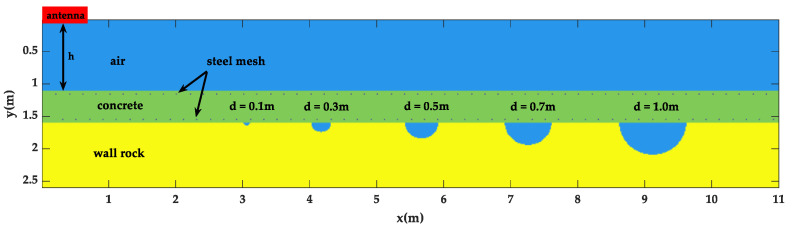
Geometric model of high-speed railway tunnel lining detection.

In this model, the air layer, the lining layer and its built-in steel mesh, the surrounding rock layer, and the semi-circular cavities of different sizes behind the lining were designed. Taking h = 1.5 m as an example, as shown in Figure 1, the simulation area was 11 m × 3.1 m, the antenna was 1.5 m away from the lining surface at 0.1 m, the lining thickness was 0.5 m, and the thickness of the surrounding rock layer was 1 m. The semi-circular cavities with diameters of 0.1 m, 0.3 m, 0.5 m, 0.7 m, and 1 m were designed at the central points (3.05,1), (4.15,1), (5.65,1), (7.25,1), and (9.1,1).

**Figure 2:**
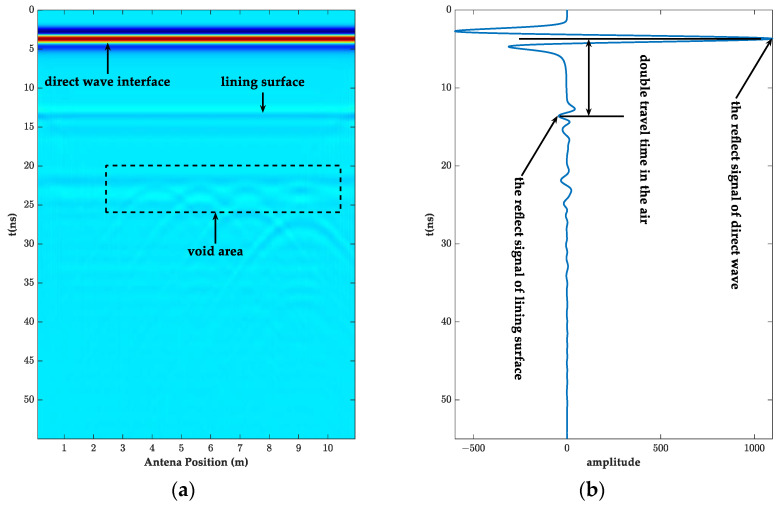
The original radar image and A-scan signal at a 1.5 m detection distance. (**a**) GPR simulation images; (**b**) A-scan signal at 1 m.

**Figure 3:**
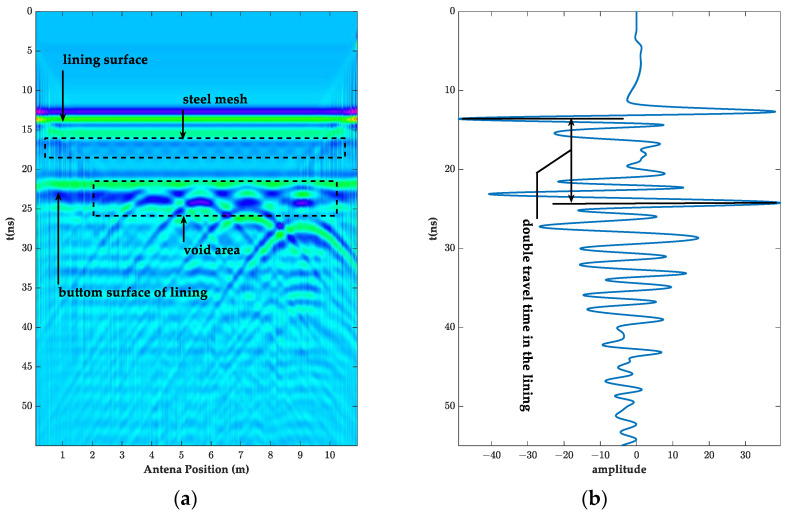
The GPR image removed direct wave and its A-scan signal at a 1.5 m detection distance. (**a**) GPR simulation images; (**b**) A-scan signal at 9 m.

The forward simulation image of the above model is shown in Figure 2. Figure 2a shows that the signal started to generate direct waves at approximately 4 ns and reached the lining surface at approximately 14 ns. As the direct wave energy largely obscured the internal information of the liner, in order to more clearly observe and analyze the change pattern of the internal signal of the liner, the direct wave processing was carried out (Figure 2a), and the results are shown in Figure 3. The reflected information on the surface and bottom of the lining can be clearly seen in the processed image, and the amplitude of the reflected waveform at the junction of the set cavity and the bottom of the lining increased significantly. The internal reflected wave increased, and the cavities, on the whole, had an obvious arc shape. However, the information at the 0.1 m diameter cavity was not very obvious. In order to verify the accuracy of the model design, the A-scan signals of the lining information at 1 m and the cavity at 9 m were extracted, respectively, as shown in Figure 2b and Figure 3b. The two-way travel time of the electromagnetic wave in the air propagation and in the lining propagation were 10 ns and 8.75 ns, respectively. The distance of the antenna from the lining surface was calculated to be 1.5 m and 0.5 m, which matched the design parameters. It also showed that the antenna could detect the lining thickness and cavity defects above 0.3 m in length at a distance of 1.5 m from the lining.

**Figure 4:**
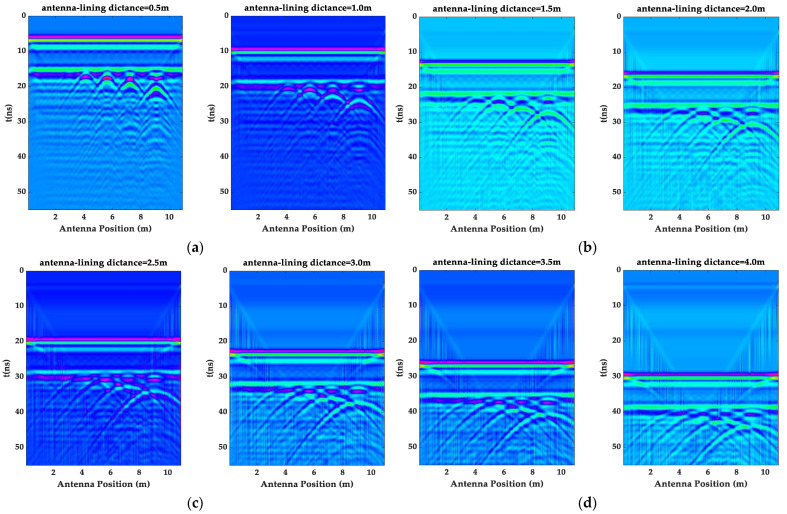
The GPR images at different detection distances. (**a**) The GPR image at a 0.5 m and 1 m detection distance; (**b**) the GPR image at a 1.5 m and 2 m detection distance; (**c**) the GPR image at a 2.5 m and 3 m detection distance; and (**d**) the GPR image at a 3.5 m and 4 m detection distance.

In order to further analyze the response characteristics of the high-speed railway tunnel images under the different detection distances, a forward simulation analysis of all the cases in Table 1 was completed. The radar forward image without the direct wave is shown in Figure 4. First, all the images clearly show the reflection information of the upper and lower interfaces of the lining. The steel mesh signal can also be seen in the images with the detection distances of 0.5 m and 1 m. Secondly, as the detection distance increased, the reflection signal of the lining interface and the void information gradually weakened. The void image information described in Figure 3 with a diameter of 0.3 m and above can be clearly observed in Figure 4.

### 2.2. The Forward Modeling of the High-Speed Railway Tunnel Interference Model and the Improvement of Its Image Interpretation Algorithm

**Figure 5:**
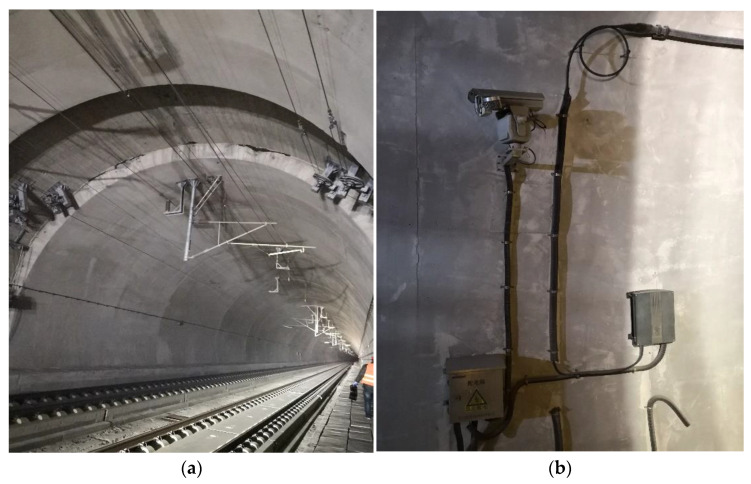
The main interference source of a high-speed railway tunnel. (**a**) Catenary supporting frames of the high-speed railway tunnel; (**b**) high-speed rail tunnel lining surface ancillary facilities.

As shown in Figure 5, since the catenary supporting frames and other ancillary structural facilities of the high-speed rail tunnel lining surface and the random noise generated by the tunnel’s electromagnetic interference clutter the GPR data, it was necessary to perform data filtering on the GPR data. For this problem, this paper used the forward simulation and improved the data interpretation algorithm for the high-speed railway tunnel interference model.

#### 2.2.1. Forward Simulation of a High-Speed Rail Tunnel Interference Model and the Improvement of Its Image Interpretation Algorithms

**Figure 6:**
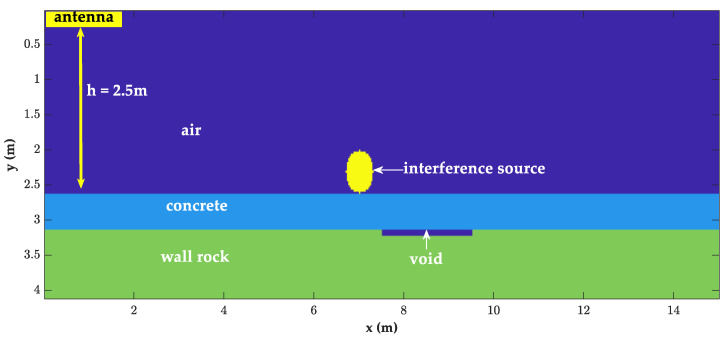
The high-speed rail tunnel interference model.

**Figure 7:**
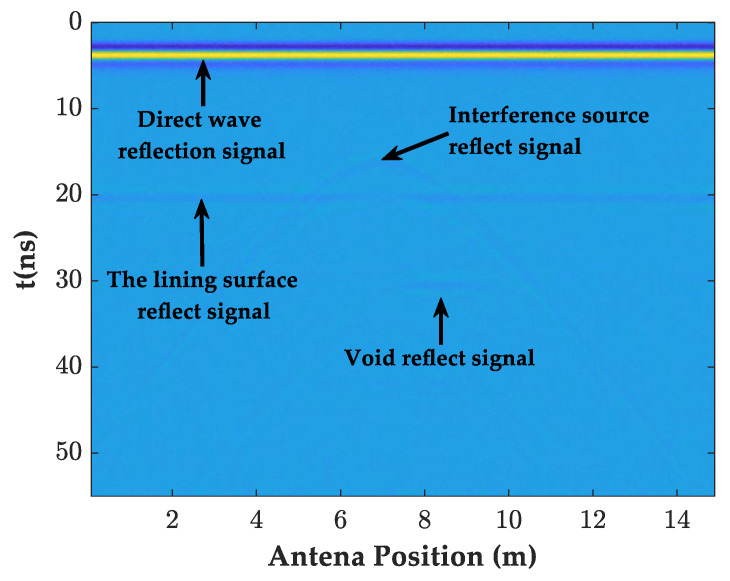
The forward GPR image with noise added.

As shown in Figure 6, this forward simulation designed a circular interference source on the lining surface and a rectangular cavity model behind the lining. The antenna started to be detected at 2.5 m from the lining surface, and the other parameters were the same as Table 1. Figure 7 shows the image after random noise was added to the GPR data.

#### 2.2.2. The Forward Modeling of F-K Filtering Based on the Butterfly Filter and Its BP Migration

**Figure 8:**
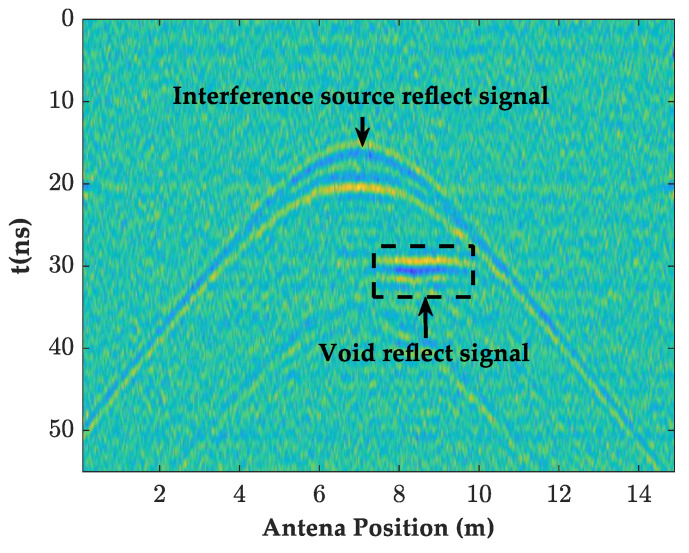
Mean and bandpass filter forward modeling image.

For the filtering of the direct waves and random noise, the traditional processing method used the mean and bandpass filtering, as shown in Figure 8. After filtering, the interference and cavity signals in the GPR images were highlighted. However, the images showed more burrs, which in turn showed that the filtering effect of the random noise was not satisfactory. In order to filter the direct wave and random noise, this paper designed a butterfly filter for filtering based on the F-K spectrum characteristics of the simulated GPR signal of the high-speed railway tunnel in order to highlight the information inside the lining.

Assuming that the received GPR echo signal of the tunnel is *f*(*t*, *x*), as shown in Formula (1), the two-dimensional Fourier transform was performed to obtain *F*(*f*, *k*).

(1) $$F(f,k)=\iint f(t,x)e^{-2\pi j(ft+kx)}dtdx$$

**Figure 9:**
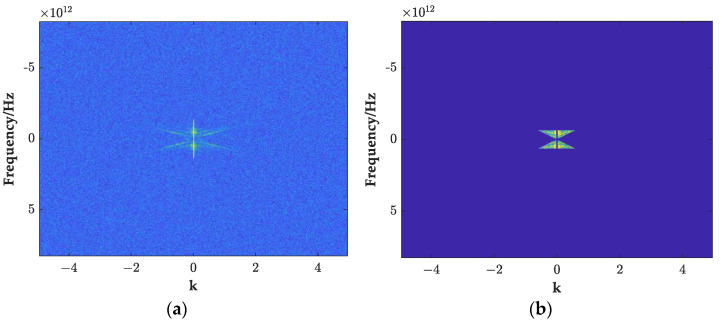
The F-K spectrum of GPR before and after filtering. (**a**) The F-K spectrum of the GPR image before filtering; (**b**) the F-K spectrum of the GPR image after filtering.

As shown in Figure 9a, in the F-K domain spectrum, the energy of the direct wave and the effective echo spectrum was mainly concentrated in the butterfly-shaped range of the wave number [−1,1], the direct wave signal was mainly concentrated in the vicinity of the wave number zero region, and the frequency of the remaining range was generated by the random noise. In this regard, this paper designed a butterfly filter, as shown in Equations (2) and (3).

(2) $$H(f,k)=\left\{\begin{array}{c} 1\;\;\;k\in effective\;zone\\ 0\;\;k\in interference\;zone \end{array}\right.$$

(3) G(f,k)=F(f,k)·H(f,k)

In the formula, *H*(*f*,*k*) is the filtering function, which is multiplied by *F*(*f*,*k*) to obtain the filtered F-K spectrum, as shown in Figure 9b. According to the designed filtering function, the random noise and direct spectrum energy of the interference area are filtered, and then the filtered data *f*(*t*,*x*) are obtained by the inverse Fourier transform of Formula (4):

(4) $$f(t,x)=\iint G(f,k)e^{-2\pi j(ft+kx)}dfdk$$

**Figure 10:**
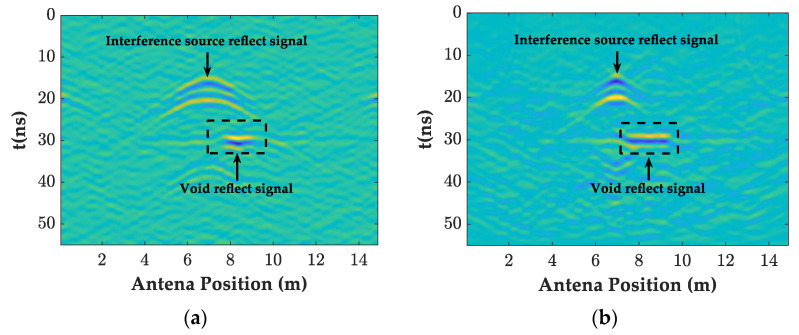
The GPR images of the different filtering methods. (**a**) F-K filtering forward modeling image; (**b**) BP migration forward modeling image.

**Figure 11:**
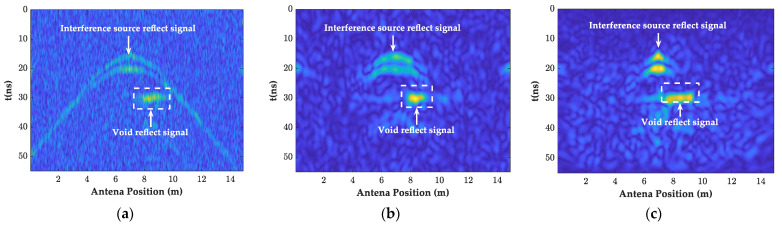
The instantaneous amplitude spectrum of the different filtering methods. (**a**) Mean and bandpass filter; (**b**) F-K filtering; (**c**) BP migration.

**Figure 12:**
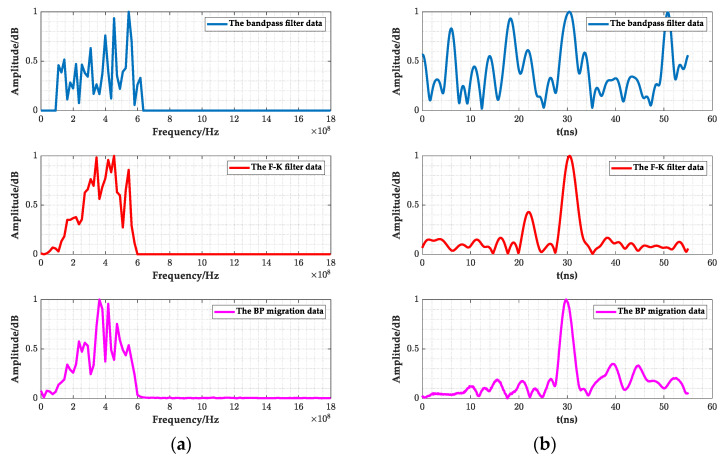
Average spectrum and instantaneous amplitude of the A-scan signal. (**a**) Frequency spectrum; (**b**) Instantaneous amplitude spectrum.

As shown in Figure 8, Figure 10a and Figure 11a,b, the filtered GPR images of the lining surface disturbance and the cavity information behind them were further revealed, the F-K filter did not show the burr representation of the random noise in the mean and bandpass filtered images, and the interface, and the cavity signals at the bottom of the lining were more obvious. As shown in Figure 12a, after further extracting the average value of all the A-scan signal spectrums at the void, it can be seen that the mean and bandpass filters at the defect had more low frequency components in the main frequency than the F-K filters, and both had varying degrees of amplitude differences. The entropy value (Hs), on the other hand, exactly reflected the overall disorder of the spectrum [22], which was calculated as follows.

(5) $$Hs=-\sum_{k=1}^{N-1}p(X_{k})lnp(X_{k})$$

In the formula, *p*(*X_k_*) denotes the probability value of the kth spectrum component. The larger the *Hs* value, the smaller the difference in the spectrum amplitude. As shown in Table 2, the entropy of the mean and bandpass filtering was 4.3, while the F-K filtering’s was 4.5, indicating that the amplitude difference between the mean and bandpass filtering was larger than that of the F-K filtering.

In further comparing the instantaneous amplitude spectrum of the two filtering methods in Figure 11b,c, it was found that the F-K filtering was free of the burr compared to the mean and bandpass filtered images. Additionally, the instantaneous amplitude of the random noise and the diffraction interference was significantly reduced. The article used the ratio of the instantaneous amplitude at the cavity defect to the average value of the sum of the instantaneous amplitudes from the beginning of the signal to the defect (amplitude ratio) *D* to describe the degree of defect accentuation, as follows.

(6) $$D=A_{t}/\bar{\sum_{k=0}^{N}A_{k}}$$

where *A_t_* denotes the instantaneous amplitude of the signal at time *t* and $\sum_{k=0}^{N}A_{k}$ denotes the average value of the sum of the corresponding instantaneous amplitude values from time 0-*t*. As shown in Figure 12b and Table 2, comparing the *D*-values of the two filtering methods showed that the F-K filtering defective signal was much more accentuated.

The strong diffraction wave signals generated by the equipment attached to high-speed rail tunnel structures can interfere with the identification of the information inside the lining. In this regard, the GPR detection field mostly adopted migration focusing [23,24], a basic inversion method to converge the diffraction energy at the original scattering point and reduce its interference with the information inside the lining. Therefore, the paper designed the orthorectified model, shown in Figure 6, with an interference source added to the liner surface and proposed a BP migration focusing algorithm suitable for dealing with the interference by migration focusing the strong diffraction wave signals. The essence of the algorithm was to superimpose the reflected waves from various points as new reflection points. The reflected wave energy from the same point was enhanced due to the in-phase coherence enhancement. The information in the A-scan of the amplitude of the received point was then focused near its apex, turning the hyperbola into a small region of strong energy, and thus increasing the lateral resolution. The core idea of the algorithm was “delay-sum”, so that the double time delay from the imaging point A to the kth channel was as follows.

(7) $$\tau_{A,k}$$

where the coordinate of imaging point *A* is *A*($x_{A}$−$z_{A}$), k represents the channel number, and *k* = 1,2,…, M. $\tau_{A,k}$ indicates the double travel time delay from imaging point *A* to the kth channel. Then, the scattering response amplitude of imaging point *A* in each channel was as follows.

(8) $$x_{A,k}=r_{k}\left(\tau_{A,k}\right)$$

where $r_{k}$ is the echo data of GPR in each channel. The response amplitude of imaging point *A* in each channel was coherently superimposed to complete the imaging of point *A*.

(9) $$E_{A}=\sum_{k=1}^{M}x_{k,A}$$

By following the above steps, the imaging of all the points was completed, and the GPR images and their instantaneous amplitude spectrum after the BP migration are given in Figure 10b and Figure 11c. Comparing Figure 10a,b, it can be seen that the interference range of the diffraction hyperbola was shortened from 5–9 m before the migration to 6–8 m, with a significant focusing effect of the interference signal. The Hs and *D*-values were increased to 4.8 and 6.6, respectively, and the rectangular cavity signal was further accentuated.

In summary, compared to the mean and bandpass filtering, F-K filtering and BP migration focusing can reduce the amplitude difference of the main frequency and improve the energy concentration of the main frequency. The *Hs* values were increased by 4.6% and 11.6%, and the *D*-values are increased by 1.2 times and 1.8 times. The random noise was suppressed, and the defect information was highlighted. It showed that the designed F-K filtering and BP migration algorithm can meet the GPR data analysis requirements for high-speed railway tunnel lining.

## 3. Analysis and Verification of Measured Data

**Figure 13:**
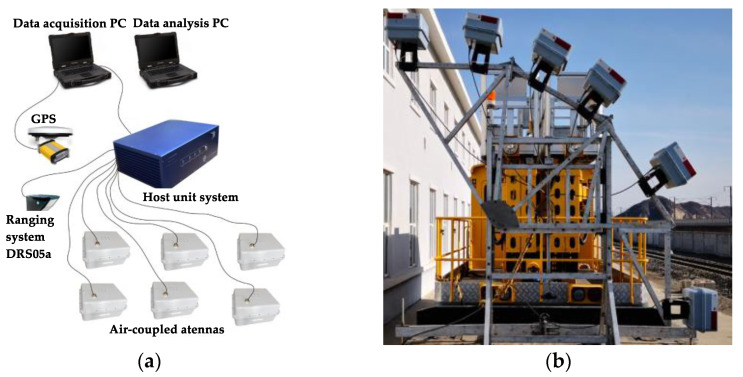
Air-coupled GPR detection system for the test. (**a**) Data collection system; (**b**) air-coupled GPR antenna group.

In order to verify the applicability of the forward simulation results, this paper selected the arch waist part of a railway double-track tunnel in North China for the field test detection. The detection length was 50 m. The test equipment was the air-coupled GPR detection system (Figure 13). It consisted of a host system, acquisition terminal, data processing terminal, mileage positioning system, and antenna group. The center frequency of the antenna was 300 MHz, the scan interval was 0.02 m, the distance between the antenna and the lining was 2.5–4 m, and the data acquisition speed was 47 km/h. The specific acquisition parameters are shown in Table 3.

### 3.1. Comparison of the Test Results

**Figure 14:**
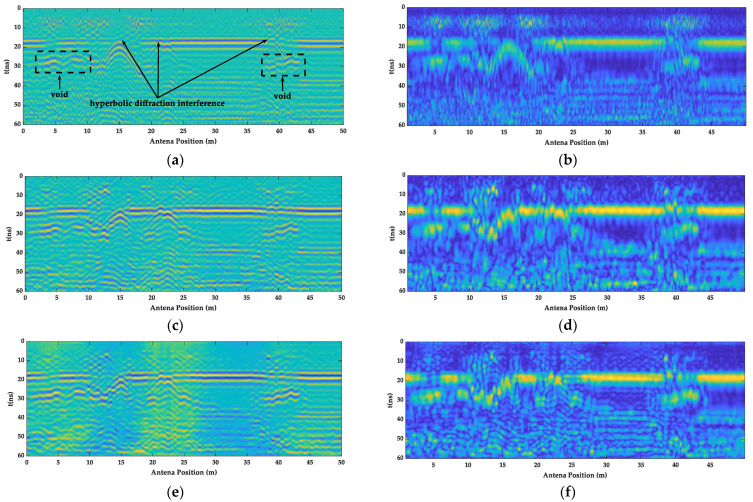
GPR measured images of the different filtering methods. (**a**) The GPR images of the mean and bandpass filter; (**b**) the instantaneous amplitude spectrum of the mean and bandpass filter; (**c**) the GPR images of the F-K filter; (**d**) the instantaneous amplitude spectrum of the F-K filter; (**e**) the GPR images of the BP migration; (**f**) and the instantaneous amplitude spectrum of the BP migration.

As shown in Figure 14, the GPR images and their instantaneous amplitude images after the mean and bandpass filtering, F-K filtering, and BP migration are given. The images from all three filtering methods reflected the lining layer information; the hyperbolic diffraction interference information generated around 15 m, 22 m, and 40 m; and the layer void information at 3–10 m and 37–44 m. Moreover, the images after F-K filtering were free from the burr of the random signal, and the lining layer and defect information were further highlighted. However, after the BP migration, the lining layer and defect information were more obvious.

**Figure 15:**
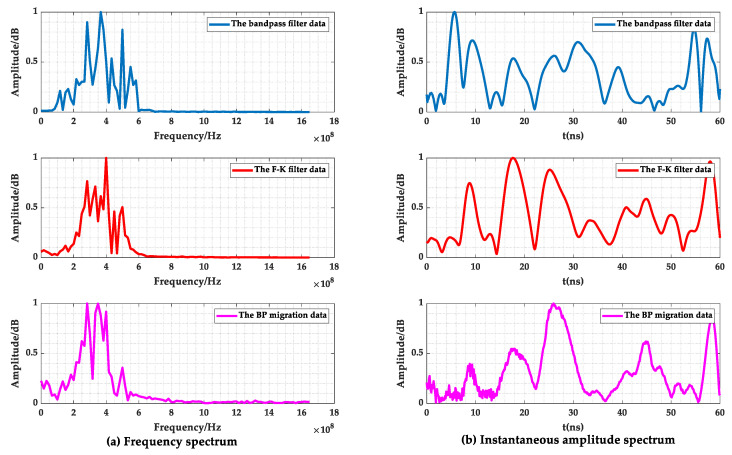
The average spectrum and instantaneous amplitude of the A-scan signal at 37–44 m.

As shown in Table 4, by comparing the entropy and amplitude ratio of the A-scan signals at 37–44 m, it can be seen that the *Hs* value of the main frequency, shown in Figure 15a, increased from 4.1 to 4.4, increasing by 2.4% and 7.3%, respectively. The amplitude difference gradually decreased. The *D* value increased from 1.5 to 3.1, which increased by 33.3% and 110%, respectively. The defect signal, shown in Figure 15b, gradually became prominent, which confirmed the performance of the GPR images information in Figure 14.

### 3.2. Verification of Drill Core Sampling

In order to verify the accuracy of the measured data, the GPR data at 5 m and 43 m were verified using drilling core sampling. The results are shown as follows.

**Figure 16:**
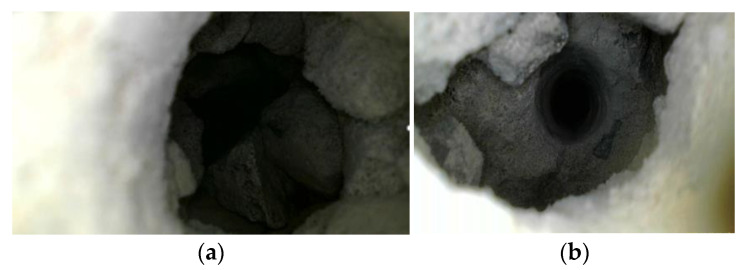
Drilling verification results photos. (**a**) The endoscopic photograph at 5 m; (**b**) the endoscopic photograph at 43 m.

For the 5 m data, the depth of the borehole was 50 cm, the thickness of the lining was 35 cm, the height of the cavity was 15 cm, and the cavity was filled with loose gravel. The endoscopic photograph is shown in Figure 16a.

For the 43 m data, the depth of the borehole was 45 cm, the bottom of the hole was the initial concrete, the thickness of the lining was 30 cm, the height of the hole was 15 cm, and the endoscopic photograph is shown in Figure 16b.

## 4. Comparative Analysis of the Air-Coupled and Ground-Coupled GPR Tunnel Measurements

**Figure 17:**
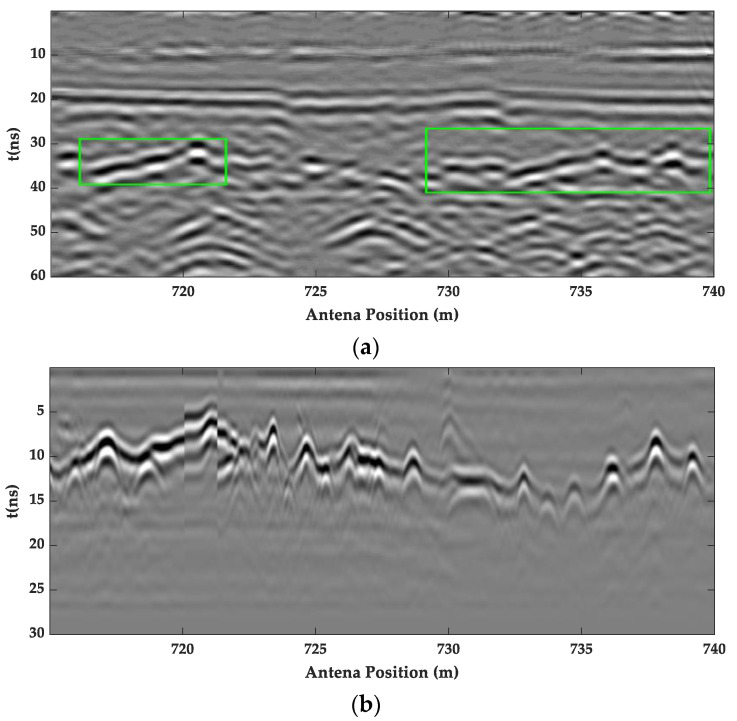
The GPR lining inspection image of the high-speed rail tunnel. (**a**) Air-coupled GPR image; (**b**) ground-coupled GPR image.

**Figure 18:**
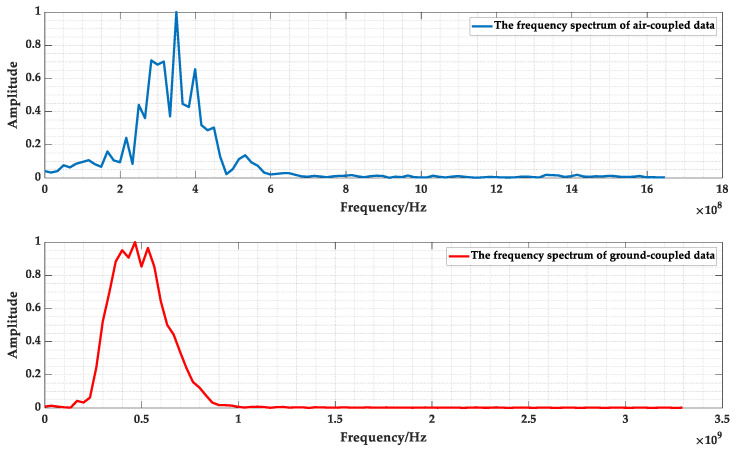
The frequency spectrum of air-coupled and ground-coupled GPR at 4 m.

In order to compare the applicability of air-coupled GPR and ground-coupled GPR in the detection of high-speed railway tunnels, the field detection of a single-hole double-line high-speed railway tunnel in a certain area of Northeast China was carried out using vehicle-mounted air-coupled GPR and ground-coupled GPR, respectively. As shown in Figure 17, it can be seen that the air-coupled and ground-coupled GPR images at 715–720 m had obvious signal characteristics of the cavity defects, and the lining interface was more obvious. As shown in Figure 18, the spectrum data from the A-scan signal extracted at 4 m showed that the amplitude difference of the ground-coupled GPR spectrum was much smaller than the air-coupled GPR spectrum, and the amplitude energy was more concentrated.

**Figure 19:**
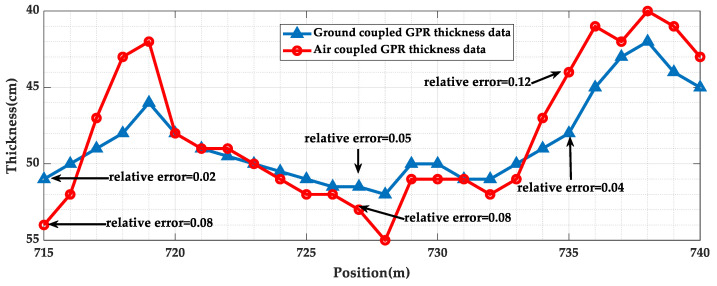
The lining thickness data.

In order to analyze the accuracy of the lining thickness measured by both methods, the lining thickness values of the measured data were extracted and interpolated and fitted, as shown in Figure 19. It was observed that both lining thickness trends were consistent, and the actual lining thicknesses of 50 cm, 49 cm, and 50 cm were obtained using drilling core samples of the lining at 715 m, 727 m, and 735 m, and the relative errors are shown in Figure 19. According to the “Regulations for Non-Destructive Testing of Railway Tunnel Lining Quality” (TB/10223-2004) [25], the relative error of the lining thickness detection using the geo-radar method should be less than 15%; thus it can be seen that the lining thickness measurement values of both testing methods were within the error tolerance, and the accuracy of the data measured using ground-coupled geo-radar was higher.

In summary, ground-coupled GPR was higher than air-coupled GPR in terms of the image clarity and detection accuracy. Therefore, the high-speed rail tunnel lining quality inspection can be combined with vehicle-mounted air-coupled GPR and ground-coupled GPR to form an initial screening census and accurate inspection of high-speed rail tunnels.

## 5. Conclusions

In order to analyze the applicability of air-coupled ground penetrating radar in high-speed railway tunnel detection, this paper analyzed and compared the signal characteristics of different filtering methods in a multi-dimensional manner using forward simulation and actual detection and drew the following conclusions.

(1) The forward simulation and real measurements of the air-coupled GPR tunnel inspection proved that air-coupled GPR with a center frequency of 300 MHz can detect the lining layer location and defects above 0.3 m, which can meet the demand of a high-speed railway tunnel lining quality inspection.
(2) In this paper, GPR forward simulations were carried out based on the characteristics of high-speed rail tunnels and the existence of interference sources, and a butterfly filter-based F-K filtering algorithm and BP migration algorithm were proposed. Using a multi-dimensional quantitative comparison with the mean and bandpass filtering algorithms, it was concluded that the improved algorithm can significantly suppress clutter, and thus highlight the deep lining signals.
(3) A comparison of the air-coupled GPR and ground-coupled GPR images and their spectrum concluded that both air-coupled GPR and ground-coupled GPR can be used for high-speed rail tunnel lining quality inspections, but ground-coupled GPR is more accurate. The advantages of these two methods complement each other, and, combined with image recognition techniques, they can provide a rapid, accurate, and comprehensive health census of high-speed railway tunnel linings.

## Figures and Tables

**Table 1:** Model design parameters.

Parameter Name	Parameter Value
Antenna-to-lining distance (m)	h = 0.5/1.0/1.5/2.0/2.5/3.0/3.5/4.0
Relative dielectric constant	ε_r1_ = 7 (concrete), ε_r2_ = 10 (rock wall)
Conductivity (S/m)	0.001
Relative permeability	1
Antenna center frequency (MHz)	300
Time window (ns)	55
Scan interval (m)	0.015

**Table 2:** Eigenvalues of the signals using different filtering methods.

Filtering Method	Hs	D
Mean and bandpass filtering	4.3	2.4
F-K filtering	4.5	5.2
BP migration	4.8	6.6

**Table 3:** Main parameters of the vehicle-mounted GPR system.

Parameters	Value	Parameters	Value
Antenna center frequency	300 MHz	Detection distance	0~4.0 m
Channel	6	Detection depth	0–2.5 m
Time window	60 ns	Working temperature	−30 °C~+70 °C
Maximum scan rate	976 scans/s	Sampling points	512/1024
Detection speed	10~120 km/h	Scan interval	0.02 m

**Table 4:** Eigenvalues of the measured signals using different filtering methods.

Filtering Method	Hs	D
Mean and bandpass filtering	4.1	1.5
F-K filtering	4.2	2.0
BP migration	4.4	3.1

## Data Availability

The original data contributions presented in the research are included in the article, further inquiries can be directed to the corresponding authors.
